# Cost evaluation, quality of life and pelvic organ function of three approaches to hysterectomy for benign uterine conditions: study protocol for a randomized controlled trial

**DOI:** 10.1186/s13063-017-2295-7

**Published:** 2017-11-25

**Authors:** Chanil Ekanayake, Arunasalam Pathmeswaran, Sanjeewa Kularatna, Rasika Herath, Prasantha Wijesinghe

**Affiliations:** 1 District General Hospital, Mannar, Sri Lanka; 2 Department of Public Health, Faculty of Medicine, University of Kelaniya, Kelaniya, Sri Lanka; 3 Centre for Applied Health Economics, School of Medicine, Griffith University, Nathan, QLD Australia; 4 Department of Obstetrics and Gynaecology, Faculty of Medicine, University of Kelaniya, Kelaniya, Sri Lanka

**Keywords:** Non-descent vaginal hysterectomy, Total laparoscopic hysterectomy, Total abdominal hysterectomy, Cost-effectiveness, Quality of life, Pelvic organ function, Randomized controlled trial

## Abstract

**Background:**

Hysterectomy is the commonest major gynaecological surgery. Although there are many approaches to hysterectomy, which depend on clinical criteria, certain patients may be eligible to be operated in any of the several available approaches. However, most comparative studies on hysterectomy are between two approaches. There is also a relative absence of data on long-term outcomes on quality of life and pelvic organ function. There is no single study which has considered quality of life, pelvic organ function and cost-effectiveness for the three main types of hysterectomy. Therefore, the objective of this study is to provide evidence on the optimal route of hysterectomy in terms of cost-effectiveness by way of a three-armed randomized control study between non-descent vaginal hysterectomy, total laparoscopic hysterectomy and total abdominal hysterectomy.

**Methods:**

A multicentre three-armed randomized control trial is being conducted at the professorial gynaecology unit of the North Colombo Teaching Hospital, Ragama, Sri Lanka and gynaecology unit of the District General Hospital, Mannar, Sri Lanka. The study population is women needing hysterectomy for non-malignant uterine causes. Patients with a uterus > 14 weeks, previous pelvic surgery, those requiring incontinence surgery or pelvic floor surgery, any medical illness which caution/contraindicate laparoscopic surgery and who cannot read and write will be excluded. The main exposure variable is non-descent vaginal hysterectomy and total laparoscopic hysterectomy. The control group will be patients undergoing total abdominal hysterectomy. The primary outcome is time to recover following surgery, which is the earliest time to resume all of the usual activities done prior to surgery. In total, 147 patients (49 per arm) are needed to have 80% power at α-0.01 considering a loss to follow-up of 20% to detect a 7-day difference between the three routes; TLH versus TAH versus NDVH. The economic evaluation will take a societal perspective and will include direct costs in relation to allocation of healthcare resources and indirect costs which are borne by the patient. A micro-costing approach will be adopted to calculate direct costs from the time of presentation to the gynaecology clinic up to 6 months after surgery. Incremental cost-effectiveness ratios (ICER) will be obtained by calculating the incremental costs divided by the incremental effects (time to recover and QALYs gained) for the intervention groups (NDVH and TLH) over the standard care (TAH) group.

**Discussion:**

The cost of the procedure, quality of life and pelvic organ function following the three main routes of hysterectomy are important to clinicians and healthcare providers, both in developed and developing countries.

**Trial registration:**

The study was registered in the Sri Lanka clinical trials registry (SLCTR/2016/020) and the International Clinical Trials Registry Platform (U1111-1194-8422) on 26 July 2016.

**Electronic supplementary material:**

The online version of this article (doi:10.1186/s13063-017-2295-7) contains supplementary material, which is available to authorized users.

## Background

Hysterectomy is the most common major gynaecological surgery, with up to 100,000 procedures performed annually in the United Kingdom [1]. The optimal route of a hysterectomy for a patient will depend on the pathological nature, uterine size, uterine descent, endometriosis and the likelihood of pelvic adhesions and adnexal masses, previous pelvic surgery and the surgeon’s preference. Although there are many methods of doing a hysterectomy the methods in mainstream practice are; total abdominal hysterectomy (TAH), non-descent vaginal hysterectomy (NDVH) and total laparoscopic hysterectomy (TLH). In some instances the best route is obvious; e.g. if the uterus is larger than 20 weeks, a TAH may be the optimal method. While in uterine prolapse, with coexistent cystocele or rectocele, a vaginal hysterectomy and repair may be the best route to deal with all the problems. However, there is a group of patients with benign gynaecological conditions for whom any one of the three main methods can be applied. It is worth finding out what is the most cost-effective method of hysterectomy in this ubiquitous group which includes a significant proportion of women undergoing hysterectomy.

Most comparative studies on hysterectomy are between two approaches; vaginal versus laparoscopic, abdominal versus laparoscopic or vaginal versus abdominal [2–6]. These studies and even systematic reviews are underpowered to detect a difference in major complications such as visceral injuries between the three main methods [2–7]. Although initial studies suggested an increased risk of urinary tract damage in TLH, the risk may not be that high now when the effects of the learning curve of laparoscopy and the evolution of the technique of TLH are considered [8]. Although direct costs appear to be high for TLH, a shorter postoperative hospital stay and lower indirect costs appear to make it more cost-effective compared to the standard TAH [4, 5, 9, 10]. There is evidence to suggest that VH appears to be less costly compared to LH according to the eVALuate trial [11]. However to date, there has been no direct comparison of costs between VH and TAH. Neither has there been any study which has attempted to shed light on the costing of hysterectomy in developing countries, where the emphasis should be on cost-effectiveness. Cost of healthcare interventions is an important aspect to consider in limited-resource settings. Although free public healthcare is available in Sri Lanka, it is important to know the cost incurred due to the huge burden on taxpayers. Therefore, direct health sector-related costs should be explored if cost-effectiveness is the ultimate aim, especially for a low- and middle-income country. Indirect costs incorporating loss of productivity are important, especially when the target population involves the population workforce, to get a wider perspective of costs incurred. Against such a backdrop, an ideal costing study would need to involve a broader societal perspective considering direct costs related to the health sector and indirect costs incorporating recovery-related costs and loss of productivity.

There is also a relative paucity of data on long-term outcomes; quality of life, urinary, bowel and sexual function in randomized controlled trials comparing surgical approaches to hysterectomy for which well-validated instruments on pelvic organ function would need to be applied in a standardised manner [2]. In addition, postoperative recovery and quality of life should be regarded as key outcomes in trials on approaches to hysterectomy for benign disease as these are the ultimate tests of effectiveness. To date there is no study which has considered quality of life, pelvic organ function and cost-effectiveness for the three main types of hysterectomy. The aim of this study is to provide evidence on the optimal type of hysterectomy in terms of cost-effectiveness by way of a three-armed randomized controlled trial (RCT) between NDVH, TLH and TAH in a low-resource setting.

### Objectives

We hypothesize that TLH and NDVH are better than TAH in terms of time to recover, quality-adjusted life years (QALYs), pelvic organ function and cost-effectiveness. It will have the added advantage of providing valuable information on health-related quality of life, pelvic organ function and costing for the commonest gynaecological major surgical procedure.

#### Primary objectives

1. To find out the time to recover after surgery following TLH, NDVH and TAH.
2. To calculate the total cost per surgery from a societal perspective for TLH, NDVH and TAH.
3. To compare the cost-effectiveness of TLH versus NDVH versus TAH.

#### Secondary objectives

1. To determine quality of life following TLH, NDVH and TAH using QALYs.
2. To evaluate the urinary, sexual and bowel function following hysterectomy.

## Methods

### Design, setting and participants

A multicentre three-armed (parallel groups) RCT was designed in accordance to the Consolidated Standards of Reporting Trials (CONSORT) recommendation for pragmatic trials [12]. The final report will follow the CONSORT 2010 guidelines as well as its extension to non-pharmacological interventions and pragmatic trials [13]. The Standard Protocol Items: Recommendations for Interventional Trials (SPIRIT) guidelines for reporting trial protocols were followed in writing this protocol (Additional file 1) [14].

**Fig. 1:**
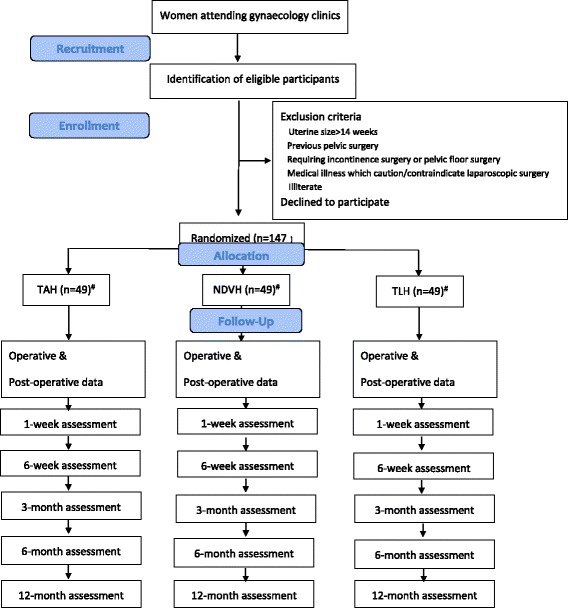
Participant flow diagram. ^#^-The required numbers are for both study centres

**Fig. 2:**
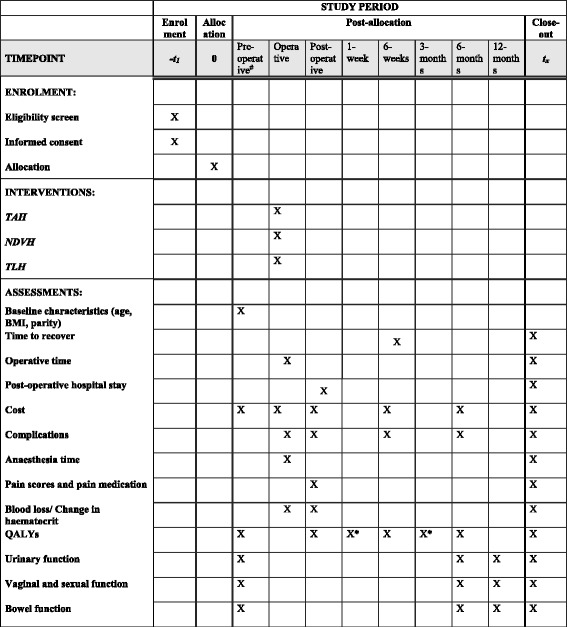
Schedule of enrolment, interventions and assessments. ^#^- preoperative assessment done 1-day prior to surgery. *-via postal assessment

The study is being done in the professorial gynaecology unit of the North Colombo Teaching Hospital, Ragama, Sri Lanka and the gynaecology unit of the District General Hospital, Mannar, Sri Lanka. Eligible participants are patients requiring hysterectomy for non-malignant uterine causes. Exclusion criteria are uterus > 14 weeks, previous pelvic surgery, those requiring incontinence surgery or pelvic floor surgery, any medical illness which caution/contraindicate laparoscopic surgery and patients who cannot read and write. Eligible patients will be aware that they will be randomly assigned to undergo one of the three procedures. The main exposure variables will be NDVH and TLH. The control group will consist of patients undergoing TAH (Fig. 1). Schedule of enrolment, interventions and assessments are shown in Fig. 2.

### Outcome measures

The primary outcome is time to recover following hysterectomy (earliest time to resume all or a combination of activities done prior to surgery; resumption of cooking, washing clothes, sexual activity and occupation). The secondary outcomes are operative time (skin to skin), postoperative hospital stay, anaesthesia time, complications (bladder injury, ureteric injury, bowel injury, convert to laparotomy, vault haematoma, blood transfusion, postoperative fever, skin wound infection, urinary tract infection, respiratory tract infection, deep vein thrombosis/pulmonary embolism, re-operation), postoperative pain and total pain medication per day per patient, QALYs, urinary, sexual and bowel function and total cost per surgery. The outcome measures are shown in Table 1.

**Table 1:** Outcome measures and study instruments

Outcomes	Study instrument
Time to recover	Patient filled data sheet at 6 weeks
Operative time	Data sheet on operative period filled by research assistant.
Postoperative hospital stay	Postoperative data sheet filled by research assistant
Cost	Direct costs based on preoperative, operative, postoperative, 6-week and 6-month data sheets filled by research assistant Indirect costs based on preoperative, postoperative, 6-week and 6-month data sheets filled by patient
Complications	Data sheet on operative, postoperative, 6-weeks and 6-months period filled by research assistant
Anaesthesia time	Data sheet on operative period filled by research assistant
Pain scores and pain medication	Data sheet on postoperative period filled by research assistant
Blood loss/change in haematocrit	Data sheet on operative and postoperative period filled by research assistant
QALYs	Paper-based EQ-5D-3L (Sinhala/Tamil) [16]
Urinary function	ICIQ-FLUTS (Sinhala/Tamil) questionnaire [18, 19]
Vaginal and sexual function	ICIQ-VS (Sinhala/Tamil) questionnaire [20]
Bowel function	Bowel symptoms questionnaire [21]

*EQ-5D-3L* EuroQoL group questionnaire on health-related quality of life, *ICIQ-FLUTS* International Consultation on Incontinence modular Questionnaire on Female Lower Urinary Tract Symptoms, *ICIQ-VS* International Consultation on Incontinence modular Questionnaire on Vaginal Symptoms, *QALYs* Quality-adjusted life years

### Sample size calculation

The primary outcome is the time to recover following surgery. A difference of 7 days was considered a clinically significant difference based on time to recover following TAH (28.1 days, SD 9.5), VH (21.3 days, SD 8.5) and LAVH (19.7 days, SD 7.5 days) in a study published in the *BJOG* by Ottosen et al. [7]. Using the mean and SD of the above data, a type 1 error of 0.01 and a power of 80% with a loss to follow-up of 20% produces a sample size of 49 patients per arm (total of 147). The reason for a type 1 error of 0.01 (two-sided) was for a Bonferroni adjustment for multiplicity to keep the overall *p* value to less than 0.05 after pairwise comparisons.

### Randomization

Block randomization in multiples of three will be carried out at each study site by opening sealed envelopes containing computer-generated block randomization numbers, with block sizes of six and nine to ensure roughly equal numbers of patients in each arm at any point in the study. The patients and medical team will not be blinded to the intervention with only the statistician performing the analyses being blinded.

### Patient selection and data collection

Eligible patients will be identified, counselled and enrolled by gynaecologists at each study centre. Before entry into the study the gynaecologist will explain to a potential patient the aims, methods, reasonably anticipated benefits and potential hazards of the study. Patient information sheets will be distributed to eligible patients and a research assistant will be available for a more detailed explanation. They will also explain to patients the potential benefit of extended long-term follow-up that would otherwise not be available and other intended benefits to the healthcare system as well as the country by participation in this research. After sufficient information, informed written consent will be obtained. Research assistants will assign enrolled participants to have either a TLH, NDVH or a TAH (Fig. 1). Patients who decline participation in the study will have standard treatment (TAH).

A preoperative questionnaire on general information, quality of life, pelvic organ function (urinary, vaginal and sexual and bowel function) and costs incurred up to surgery would be filled in by the patient while another on relevant medical information would be filled by a research assistant a day prior to surgery. Standard techniques of TLH, TAH and NDVH would be followed [15, 16]. The decision to convert to laparotomy at TLH or NDVH would be at the discretion of the primary surgeon.

Data on operative time, complications, instruments and medications used and staff allocation during surgery will be entered into a data collection sheet immediately after surgery. During the postoperative period the following information will be collected by a research assistant using a datasheet; complications, pain scores and analgesic medication and investigations. A patient-filled questionnaire will obtain costs incurred by the patient due to hospital admission. The health utility scores based on paper-based EuroQoL group health-related quality of life (EQ-5D-3L) questionnaires will be estimated on day 2 and 1 week post-surgery. (A self-addressed stamped envelope along with an EQ-5D-3L form will be provided to the patient on discharge with instructions to mail the 1-week assessment).

Patients will be reviewed at 6 weeks in the gynaecology clinic and information on time to recover, health utility scores, hospital admissions, complications and investigations will be collected. Health utility scores will be estimated at 3 months by sending an EQ-5D-3L form via post along with a stamped self-addressed envelope. Patients will be reviewed at 6 months in the gynaecology clinic and information on health utility scores, pelvic organ function in addition to any complications or readmissions would be obtained. At the 1-year visit, information on pelvic organ function will be collected. The timing of assessments for each outcome is shown in Table 1.

### Description of study instruments

The validated paper-based Sinhala and Tamil EQ-5D-3L questionnaire will be used to obtain health utility scores. The EQ-5D-3L is a generic multi-attribute utility system, which assess health-related quality of life on five dimensions (mobility, self-care, usual activities, pain/discomfort, anxiety/depression) [17]. Based on the responses a woman will be placed in to one of 243 (3^5^) mutually exclusive health states, and a value for each of these has previously been estimated with ‘0’ being equivalent to dead and ‘1’ being equivalent to perfect health on a utility scale based on interviews. EQ-5D 3L questionnaires will be administered 1 day preoperatively and on postoperative day 2, 1 week, 6 weeks, 3 months and 6 months postoperatively. Each woman’s QALYs will be calculated using the utility score and the time spent on the particular health state. A utility value table specific to Sri Lanka will be used to calculate QALYs [18].

Urinary and sexual function will be assessed by the validated Sinhala and Tamil translations of International Consultation on Incontinence Modular Questionnaire on Female Urinary Tract Symptoms (ICIQ-FLUTS) and Incontinence Modular Questionnaire on Vaginal Symptoms ICIQ-VS which were obtained from the International Consultation on Incontinence (ICI) [19–21]. Bowel symptoms will be assessed by a questionnaire based on a study done by Tharkar et al. [22]. These questionnaires will be used preoperatively, 6 months and up to 1 year post-hysterectomy to detect changes in pelvic organ function.

Postoperative pain evaluation will be done using numeric pain rating scale (NRS) [23], asking the patient to indicate the intensity of current, best, and worst pain levels over the past 24 hours on a scale of 0 (no pain) to 10 (worst pain imaginable). The average of the three ratings; average, best, and worst will be used to represent the patient’s level of pain over the previous 24 hours. To allow direct comparison of pain medication a modified World Federation of Societies of Anaesthesiologists (WFSA) analgesic ladder will be used [24].

### Data security and cleaning

The completed questionnaires and data collection sheets will be collected and entered locally in an ongoing computer database at each centre. Data will be stored in password-protected folders. An independent data monitoring committee comprising of staff from the computer centre of the Faculty of Medicine, University of Kelaniya will undertake regular checks and monitoring to ensure compliance and enhance accuracy at both centres.

### Compliance issues

Participants can exit the trial at any given point. Exit is defined as exiting the trial altogether or wanting another procedure different from the one allocated by randomization. If they exit after randomization but prior to surgery it will be mentioned but they will not be included in the analysis. However, if they wish to exit the trial after surgery, we will retain their data and their permission to access healthcare records unless consent for these activities is explicitly withdrawn. We will also actively try to minimize actual loss to follow-up by contacting patients through available contact information and access to information in hospital. We feel the rate of non-compliance will be low as well-motivated women will participate in a study of this nature. We also feel that it will be the same for participating gynaecologists as competent and well-motivated individuals who want to help answer the research question were chosen. Divergence from a pre-specified trial arm will be documented with reasons and included in the analysis of the originally allocated trial arm.

### Data analysis

All randomized patients will be included in the analyses. A single principal analysis will be done comparing the three arms (TAH, NDVH and TLH) 12 months after the last woman has had her operation. The fact that the trial will be conducted at two centres will be taken into account during data analysis.

A Kaplan–Meier survival analysis will be done for the primary outcome which is time to recover following hysterectomy. The end-point is the actual time to recover. It will be assessed and documented at the 6-week clinic visit. Patients who have not recovered by 6-weeks will be followed-up beyond 6-weeks to detect complications and the actual time to recover. Post hoc pairwise analysis between TAH, NDVH and TLH will be done using COX regression.

Other outcomes that need follow-up; QALYs, urinary, vaginal and bowel functions will also be analysed using Kaplan Meier survival analyses. The end point for QALYs is 6-months while for urinary, vaginal and sexual function it is 1-year. Patients who do not complete follow-up in these secondary outcomes will be right censored up to their respective end-points. Post hoc pairwise analysis between TAH, NDVH and TLH will be done using COX regression. The other outcomes; operative time, anaesthesia time, postoperative hospital stay, cost, pain scores and blood loss will be analysed using Kruskal-Wallis test with post hoc comparisons. The complications will be described for each category. A fully specified Statistical Analysis Plan will be provided before opening the database.

### Economic evaluation

The economic evaluation will take societal perspective and will include direct costs related to utilization of healthcare resources in the hospital and indirect costs which are borne by the patient. A micro-costing approach will be adopted to calculate utilization of hospital resources from the time of presentation to the gynaecology clinic up to six months after surgery. Incremental cost-effectiveness ratios (ICER) will be obtained by calculating the incremental costs divided by the incremental effects (time to recover and QALYs gained) for the intervention groups (NDVH and TLH) over the standard care (TAH) group.

### Resource use and costs

Direct costs would include surgical costs, clinic costs, investigation costs and care during hospital stay. Surgical costs will consider staff cost, equipment cost, anaesthetic costs and cost of basic utilities; water, electricity and building cost. Clinic costs will entail staff cost, equipment cost and cost of basic utilities for clinics during the preoperative and postoperative period. Investigation costs will include laboratory and imaging costs for preoperative period and postoperative period. Information on routine investigations; full blood count, blood urea, serum electrolytes, urine full report, ECG, chest X-ray, transvaginal ultrasound scan (TVS) and any other additional investigation would be collected from clinic and in-ward medical records by the data collector on to a data sheet. The cost of hospital stay will include staff costs, cost of medicines and basic utilities. Information on healthcare costs were obtained from the medical supplies division of the Ministry of Health, Sri Lanka. Information on labour costs and basic utilities were obtained separately for each centre. Indirect costs will consider costs of additional investigations and medicines paid for by the patient, travel costs to and from the hospital/clinic, cost for carers and productivity losses for the patients and families and will be obtained by patient-filled questionnaires.

### Organisational structure

The study office is in the professorial obstetrics and gynaecology unit of the faculty of medicine, University of Kelaniya. It communicates with North Colombo Teaching Hospital-Ragama and District General Hospital, Mannar on specific issues. Each site is responsible for randomization and data collection. Monthly meetings are held in the study office with the participation of the involved gynaecologists at North Colombo Teaching Hospital, Ragama and the lead gynaecologist from District General Hospital, Mannar to ensure the smooth running of the trial. Six monthly updates will be provided to the Faculty of Graduate Studies of the University of Kelaniya and the Sri Lanka Clinical Trials Registry (SLCTR) along with yearly progress reports to the funding agency, National Research Council (NRC) of Sri Lanka. The study is supervised by its project management group, which consists of a representative of the grant agency and representatives from the study centres.

### Local organisation in centres

Each centre will have a lead gynaecologist who will be the point of contact for that centre whose responsibilities will be: to establish the study locally, obtaining the required regulatory approval; to appoint and train a local research assistant; to inform all relevant local staff about the study (e.g. other consultant gynaecologists, junior medical staff, research assistants and ward staff); take responsibility for complications of the study locally; and notify the study centre of any unexpected complications due to the study. Fortnightly meetings involving gynaecologists, research assistants, junior medical staff and paramedical staff will be held at each local centre. The involved gynaecologists will identify eligible patients, explain the different surgery options to them, and ensure that study documentation has been provided and that informed consent has been obtained.

Each centre will have a local research assistant to organise recruitment of patients to the study. The responsibilities of the research assistant will be to: ensure that each participating patient has received the patient information leaflet and has given informed written consent, ensure that each participant has received all self-administered questionnaires, collect all relevant medical data on to the data sheet, keep a log of all eligible women and their recruitment status, maintain a register of all recruited patients, contact participants at relevant assessment points to obtain required data (at 1 week, 6 weeks, 3 months, 6 months and 1 year), systematic filing of study documents, data entry on to the computer database, notify the local lead gynaecologist of any problem or complication and inform local staff of progress of the study. In order to improve recruitment, research assistants will identify any eligible women in the ward who may have been missed at the gynaecology clinic.

### Research governance

The computer unit of the Faculty of Medicine, University of Kelaniya will help to provide centralised database support. The principal investigator will ensure that sufficient mechanisms are in place for monitoring the quality of the study with timely routine reports of adverse effects. The trial will comply with the UK Data Protection Act 1998. The trial statistician will have access to the final trial dataset and will manage access rights to the dataset. If compliance with legal, data protection and ethical guidelines are met anonymised trial data will be shared with other researchers once all planned data analysis is complete.

### Safety monitoring and premature termination of study

All surgeries will be done by consultants competent in the relevant type of hysterectomy, i.e., for an example all hysterectomies will be done by specialists who have operated on a minimum of 25 procedures. Any complication would be managed as per standard guidelines and necessary multidisciplinary input sought if required. All complications will be reviewed by an independent clinical review panel comprising three experienced gynaecologists. A list of all the possible complications will be made available at each site. It is the responsibility of the researchers and all other grades of staff to inform the clinical review committee of any complication through incident report forms, which are available in each setting. The clinical review panel will assess if the complications in the TLH or NDVH group exceed in amount or severity compared to the standard treatment (TAH group). If so, the disadvantaged arm of the study will be terminated.

### Main ethical issues

All three hysterectomy methods described above are standard treatment modalities in operative gynaecology. Therefore, we believe that we are not intentionally harming patients by including them in this clinical trial.

### Dissemination policy

The results of the study will be submitted for publication to peer-reviewed medical journals and presented at symposia regardless of the outcomes.

## Discussion

Studies in low-resource settings are hampered by financial limitations and feasibility in terms of time and collection of good quality accurate data. This protocol considers many relevant aspects concerned with the comparison of total abdominal, non-descent vaginal and total laparoscopic hysterectomy in one study for a lower-middle-income country where evidence on surgical outcomes especially in the form of randomized controlled trials are hard to come by. We believe these reasons make this protocol appealing for other researchers in similar settings.

As this is a multicentre study the involvement of multiple surgeons is inevitable and this can raise the issue of various skill levels and affect the outcome of surgery rather than a single operator. Although the issue of multiple surgeons is a limitation to the study itself, the generalisability of the results to the rest of the country would be more in our design of a pragmatic multicentre study involving multiple surgeons for each method rather than a single-centre study with a single surgeon for each method. In a study of this nature, it would not be possible to blind the patients nor the surgeons as the scars are distinct in each method. Although this is a limitation it is unavoidable and only the statistician will be blinded. The use of objective questionnaires, for patients’ as well as for assessors will also help to minimize bias [12].

The primary outcome, of time to resume usual activities, could be subject to recall bias. As it is a patient-reported outcome it has the advantage of being the best estimator of convalescence in the eyes of patients despite being subjective. Pelvic organ function is also assessed through patient-filled questionnaires as there is no objective method of assessing postoperative pelvic organ function. Although patient-reported outcomes can be subjective, validated questionnaires such as the ones used in this study make these assessments more valid and reliable. These questionnaires are the only available, non-invasive and generically applicable tools that can be used in low-resource settings.

Major complications (bladder, ureteric, bowel) are relatively rare in hysterectomy for benign surgery and as such a very large sample size would be needed if it was selected as the primary outcome. This is not feasible in our context and this study is underpowered to detect these problems. However, it could be assumed that if these complications were to occur, it would in some way be reflected by the time to recover and QALYs as these measures will show the overall quality of life of the individual concerned. Assessment of menopausal symptoms post-hysterectomy would need serum FSH levels before and after surgery if it is to be assessed in a quantitative manner, but it is not available in the public sector in Sri Lanka. Post-hysterectomy vaginal vault prolapse is a rare long-term complication following hysterectomy which would require follow up at least of up to 10 years, which is not feasible in our setting.

Despite these disadvantages, this randomized controlled trial will provide level 1 evidence regarding the three main methods of hysterectomy in terms of operative time, postoperative hospital stay, cost, convalescence, sexual dysfunction, bladder, bowel function and QALY’s. It will also establish a quantifiable and reproducible method of accessing outcomes for benign gynaecological surgery.

Although exact figures for rates of hysterectomy are difficult to obtain for developing countries it can be assumed to be very high due to the non-availability of levonorgestrel intrauterine system and other medical alternatives due to financial constraints, and as such TAH is often offered by default. This study will shed light on whether it is indeed the most appropriate in terms of health- related quality of life and cost-effectiveness. If it is not, i.e. if NDVH or TLH is better than TAH, it will suggest a change in attitude of gynaecologists, resource allocation from the Ministry of Health and even postgraduate training to lay more emphasis on alternative methods of hysterectomy.

There is very limited information on costs related to healthcare interventions in Sri Lanka possibly due to a misconception of *‘free health’*. The societal viewpoint in calculating costs in our study will generate an accurate picture of both direct hospital-related and indirect patient-related costs. The micro-costing approach used in this study is reported to be the most accurate method in calculating hospital cost [25]. Therefore, this study will provide valuable information on costing a surgical intervention, the methodology of which will be helpful across the board for other specialities as well as similar scenarios in other developing countries.

Sri Lanka is a lower-middle-income country, and as such it is important for us to enquire in to cost to ensure that maximum value for money is obtained with the limited resources at hand. Therefore, cost-effectiveness should be the benchmark to assess healthcare interventions in such a backdrop. This study will shed light on the optimum type of hysterectomy in terms of cost-effectiveness considering both the clinicians and patients’ perspectives.

### Trial status

The first patient was randomised to the trial on 1 August 2016. The trial is currently ongoing in the two centres and according to current rates of recruitment we envision the trial to complete recruitment by September 2017 with the last participant follow-up expected in September 2018.

Protocol version number 03: 26 July 2016

## Additional file

Additional file 1: SPIRIT checklist. (DOC 124 kb)

## Abbreviations

EQ-5D-3L: EuroQoL group questionnaire on health-related quality of life
ICIQ-FLUTS: International Consultation on Incontinence modular Questionnaire on Female Lower Urinary Tract Symptoms
ICIQ-VS: International Consultation on Incontinence modular Questionnaire on Vaginal Symptoms
NDVH: Non-descent vaginal hysterectomy
POP-Q: Pelvic organ prolapse quantification
QALYs: Quality-adjusted life years
RCT: Randomized controlled trial
TAH: Total abdominal hysterectomy
TLH: Total laparoscopic hysterectomy
